# Brief Report: Feasibility of the Probabilistic Reversal Learning Task as an Outcome Measure in an Intervention Trial for Individuals with Autism Spectrum Disorder

**DOI:** 10.1007/s10803-021-05288-y

**Published:** 2021-09-23

**Authors:** Lauren M. Schmitt, John A. Sweeney, Craig A. Erickson, Rebecca Shaffer

**Affiliations:** 1 Cincinnati Children’s Hospital Medical Center, Cincinnati, USA; 2 College of Medicine, University of Cincinnati, Cincinnati, USA

**Keywords:** Cognitive flexibility, Reversal learning, Autism spectrum disorder, Outcome measurement

## Abstract

Cognitive flexibility deficits are a hallmark feature of autism spectrum disorder (ASD), but few evidence-based behavioral interventions have successfully addressed this treatment target. Outcome measurement selection may help account for previous findings. The probabilistic reversal learning task (PRL) is a measure of cognitive flexibility previously validated for use in ASD, but its use as an outcome measure has not yet been assessed. The current study examined the feasibility, reproducibility, and sensitivity of PRL in a within-subjects trial of *Regulating Together*, a group-based intervention targeting emotion regulation. We demonstrated the PRL is highly feasible, showed test–retest reproducibility, and is sensitive to detect change following the intervention. Our findings demonstrate the PRL task may be a useful outcome measure of cognitive flexibility in future intervention trials in ASD.

## Introduction

Individuals with autism spectrum disorder (ASD) frequently display cognitive inflexibility and rigidity. Deficits in cognitive flexibility in ASD are exacerbated under conditions of uncertainty and high emotional saliency, and are associated with worse outcomes including poor academic success, aggressive behavior, impaired emotion regulation (ER), and troubled social relationships (Bos et al., 2019; Cai et al., 2018; D'Cruz et al., 2013; Lawson et al., 2014; Mazefsky & White, 2014; Memari et al., 2013; Schmitt et al., 2019; Van de Cruys et al., 2017; Visser et al., 2014). Currently, there are few behavioral interventions that target cognitive flexibility in ASD. Available studies have demonstrated mixed efficacy in improving cognitive flexibility based on both parent-report and performance-based outcome measures (for positive findings: Kenworthy et al., 2014; Saniee et al., 2019); for negative findings: (de Vries et al., 2015; Fisher & Happé, 2005; Yerys et al., 2019).

Though there is a clear need for better interventions targeting cognitive flexibility in ASD, previous equivocal findings likely are due, in part, to inadequate outcome measures focusing specifically on this cognitive/behavioral deficit. For instance, traditional neuropsychological tests, like the Wisconsin Card Sorting Task, often have been used in ASD intervention studies to assess cognitive flexibility despite the fact that these tests measure multiple cognitive constructs simultaneously (Kenworthy et al., 2008). Furthermore, parent-report measures used as cognitive flexibility outcome measures in previous intervention studies often were developed for academic or outpatient clinical settings to describe general problems related to cognitive flexibility deficits over a wide time window rather than being sensitive to detect smaller changes over a short period of time (Brugha et al., 2015; Grzadzinski et al., 2020). The availability of novel neurocognitive strategies for evaluating treatment outcome in this particular behavioral domain would address the need for improved outcomes measures for intervention research in neurodevelopmental disorders. Therefore, the development of quantitative measures associated with targeted behavioral domains of interest is an important effort for advancing ASD-related intervention research (Budimirovic et al., 2017).

The probabilistic reversal learning task (PRL) is a translational measure of cognitive flexibility that our group has previously validated for use in ASD (D'Cruz et al., 2013; Schmitt et al., 2019). PRL has shown feasibility and sensitivity to change in pharmacological intervention studies in mouse models of ASD (Amodeo et al., 2012, 2014). However, this is the first time, to our knowledge, that PRL has been used as an outcome measure in a behavioral intervention trial for individuals with ASD. This Brief Report examines the feasibility of PRL as outcome measure following a pilot trial of *Regulating Together *(*RT*), a group based behavioral intervention targeting ER in individuals with ASD aged 8–18 years (Shaffer, Under review; Shaffer et al., 2019). We hypothesized that PRL would be highly feasible and acceptable to individuals with ASD. We also predicted that PRL would demonstrate test–retest reliability with the 5-week lead in period to intervention as well as sensitivity to change based on improved performance from baseline to post-intervention.

## Methods

### Participants

Sixty-two participants (88% male) with a DSM 5 diagnosis of ASD aged 8–18 years completed the intervention trial across six rounds of RT (5 rounds of Child for 8–12 years old, 4 rounds of Adolescent for 13–18 years old). The sample included 78.8% White, 5.8% African American, 5.8% Asian, 1.9% Native Hawaiian/Pacific Islander, 3.8% Other race, and 8.7% had Hispanic ethnicity. 5% chose not to report racial/ethnic backgrounds. Co-occurring diagnoses included 60.4% ADHD, 58.7% Anxiety Disorders, 21.7% Depression, 17.4% Intermittent Explosive Disorder, 15.2% Insomnia, 25.5% OCD, 19.6% Oppositional Defiant Disorder, and 6.7% PTSD. At least one psychotropic medication was taken by 45% of participants. During Screening, all participants completed the Autism Diagnostic Observation Schedule, Second Edition (ADOS-2) and Wechsler Abbreviated Scale of Intelligence, Second Edition (WASI-II) to confirm diagnosis and assess intellectual ability. Individuals were excluded if they did not meet DSM 5 criteria for ASD, were not able to communicate with complex speech (as indicated by Module 3 or 4 of the ADOS-2), did not have at least one caregiver able to participate in the study, English was not their or their caregiver’s primary language, or had an IQ < 60. Participants had to score ≥ 10 on either the Irritability or Hyperactivity subscale of the *Aberrant Behavior Checklist, Second Edition *(*ABC-2*) (Aman & Singh, 2017) to be included in the intervention study. The study was approved by the local IRB in accordance with the Declaration of Helsinki. All caregivers provided informed written consent and participants provided oral assent when appropriate.

### Task Description

**Fig. 1:**
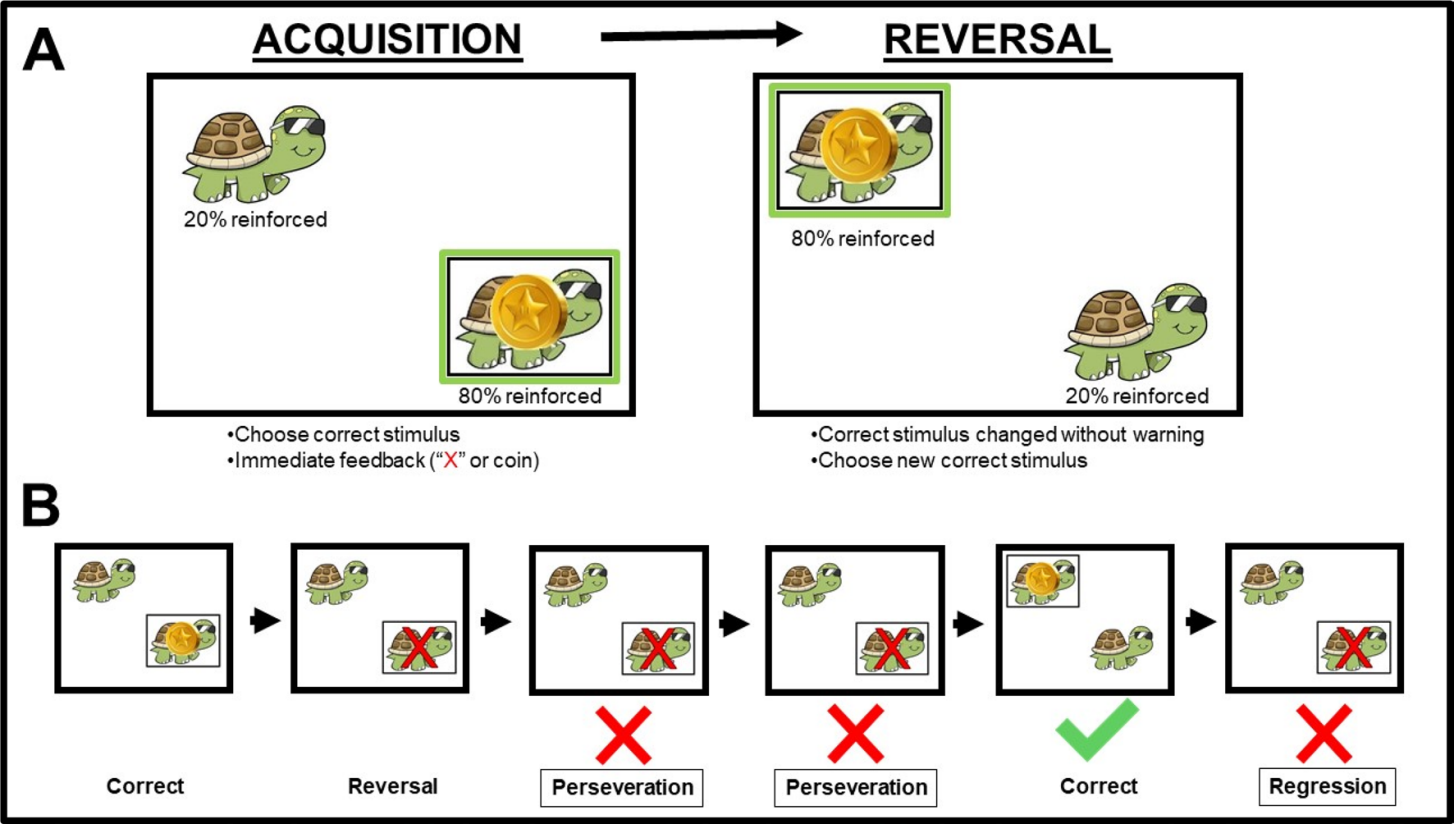
Schematic of the Probablistic Reversal Learning task (**A**) and error types (**B**)

During the *Probabilistic Reversal Learning Task* (*PRL*), participants were instructed to choose one of two identical stimuli (i.e., animals) positioned in different locations on the screen (D'Cruz et al., 2013; Schmitt et al., 2019). Participant behavior was reinforced (i.e., coin) on 80% of correct responses and on 20% of incorrect responses (Fig. 1). During the acquisition phase, participants chose one of two stimulus locations until they identified the correct location on 8 of 10 consecutive trials. Then, they proceeded to the reversal phase in which the correct location is switched without warning, and participants had to identify the new correct location on 8 of 10 consecutive trials. Testing was discontinued if they did not reach criterion within 50 trials on either phase. Participants completed two practice tests to establish test comprehension. We computed total number of trials to reach criterion and number of errors after reversal (i.e., selecting the incorrect location). Two different error types were computed during the reversal phase: perseverative errors, or *continuing* to choose the previous correct location following reversal to the new correct location, and regressive errors, failing to maintain the new correct location and *returning* to the previous correct location.

### Intervention

Briefly, RT is a 10-session, 5-week group-based ER intervention for individuals with ASD, with nearly identical curricula for child (8–12 years) and adolescent age groups (13–18 years). RT targets ER by utilizing multiple evidence-based intervention strategies from applied behavior analysis, cognitive-behavioral therapy, mindfulness and acceptance, and dialectical behavior therapy (Shaffer, Under review; Shaffer et al., 2019). Caregivers participate in a concurrent group to learn the same material, general behavioral management, and coaching strategies to help reinforce new skills in their children. Each session is 90 min and focuses on one primary topic related to ER, using a variety of techniques to teach and practice material. While the intervention targets ER, we included specific curricula focused on enhancing cognitive flexibility by building vocabulary around flexibility, increasing awareness of one’s own inflexibility, and applying specific problem solving strategies to increase flexibility via group exercises and games, individual practice and repetition, and homework to rehearse and practice skills. Full details of the development of RT and its curriculum are available elsewhere (Shaffer et al., 2019). Our pilot intervention study included five study visits: Screen, Baseline/Treatment Start, Treatment End, Treatment Follow-Up 1 (5-week follow-up), and Treatment Follow-Up 2 (10-week follow-up) in which individuals with ASD and their primary caregiver completed a battery of outcome measures.

### Statistical Analysis

To assess feasibility, we examined completion rates at each time point and identified reasons for failed completion. To assess test–retest reliability of PRL variables, we calculated inter-class correlations (ICC) for Screen and Baseline time points, using data from subjects who successfully completed the task at both time points (Koo & Li, 2016). To assess the task’s sensitivity to change, we examined changes in behavioral performance on PRL primary variables following intervention using repeated measures ANOVAs with within-subjects variable Time Point (Screen vs Baseline vs Treatment End vs 5 Week Follow-up vs 10 Week Follow-up) and PRL variables. Age Group was added as a between-subjects factor (Child vs Adolescent) in secondary ANOVA models in order to identify potential age-related effects. Due to the preliminary nature of our analysis, we conducted planned comparisons to probe differences between Baseline and the three post-intervention visits, and we used Fischer’s Least Significance Difference (LSD) test as a liberal approach to correction for multiple comparisons.

## Results

### Feasibility and Acceptability of PRL

**Fig. 2:**
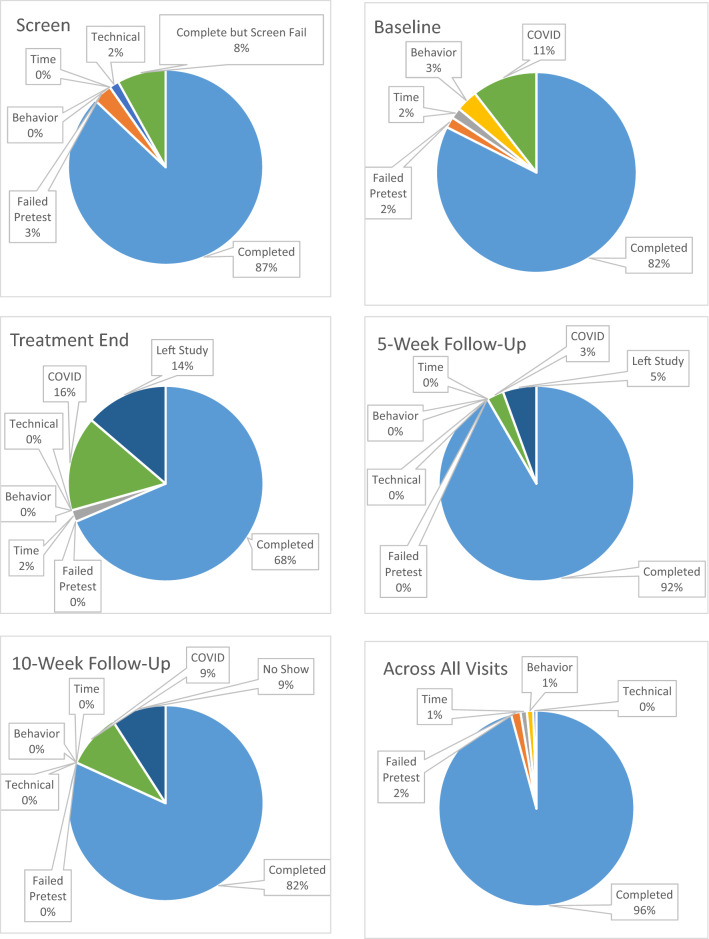
Completion rates (in order of appearance) across screen, baseline/treatment start, treatment ned, 5-week follow-up, 10-week follow-up, and all visits

Completion rates and reason for failures for each study visit are found in Fig. 2A–E. Briefly, at the screen visit, one participant did not complete testing due to technical difficulties (1.6%) and two participants attempted, but failed the pre-test (3.2%). Thus, a total of 59 out of 62 (95.2%) completed PRL testing. Following screen, five individuals did not meet inclusion criteria and six individuals were not able to complete any additional in-person visits after baseline testing due to the COVID-19 pandemic. Thus, at the baseline visit, there was a total of 51 participants among which two participants did not complete testing due to behavior (3.9%), one due to time limitations (2.0%), and one failed the pretest (2.0%). The remaining 47 (92.2%) completed PRL at baseline. Seven individuals left the study prior to intervention completion (13.7%), eight individuals were unable to complete testing following intervention completion due to the COVID-19 pandemic (17.0%). Thirty-six participants were available for testing at intervention end. Of these, 35 participants completed the task (97.2%); one could not complete due to time restrictions within the study visit (2.8%). At the 5-week follow-up visit, one person could not complete due to COVID-19 and two participants canceled their testing appointments. However, 100% of the participants who participated in the 5-week follow-up visit completed the task. At the 10-week follow up, three participants were impacted by COVID-19 and three participants canceled their visit; however, 100% of the 29 individuals were able to attend visit completed PRL testing.

In summary, of the 215 possible testing sessions across participants, only three sessions were not completed due to participant behavioral challenges (1.4%), three due to time constraints (1.4%), two due to failing the pre-test (0.9%), and one due to technical issues (0.5%; Fig. 2F). Three distinct participants demonstrated challenging behavior in the testing appointment that limited their ability to complete PRL, all of whom were 8 years old. For example, it was noted that one participant ran out of the room and into the parking garage during the session. However, none of the three participants showed behavioral challenges that limited testing at subsequent visits. Two different participants failed the pre-test, one during their screen visit and one during their baseline visit. Each were in the Adolescent group and had IQs in the borderline range. Yet, both participants were able to pass the pre-test and continue onto PRL during subsequent visits. Overall, we collected data at 206 (95.8%) of possible sessions.

Acceptability was not formally assessed, but based on behavioral observation the majority of participants demonstrated tolerability. The three participants described above who demonstrated behavioral challenges during PRL were observed to show similar behavior throughout that specific testing appointment. Thus, we believe the behavior was not specifically related to unacceptability of this task, especially since challenging behaviors were not observed at subsequent visits.

### Test–Retest Reliability of PRL

Our estimated test–retest reliability of number of trials to criterion was ICC 0.73, with 95% CI (0.50, 0.86). With regard to errors, perseverative errors had ICC 0.70 (95% CI 0.47, 0.85) and regressive errors had ICC 0.66 (95% CI 0.40, 0.82).

### Behavioral Performance During PRL

**Fig. 3:**
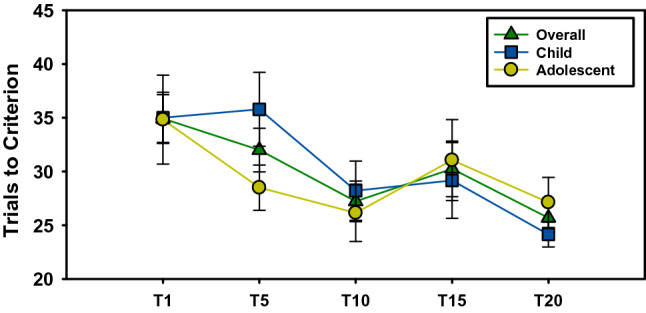
Total trials to criterion

**Fig. 4:**
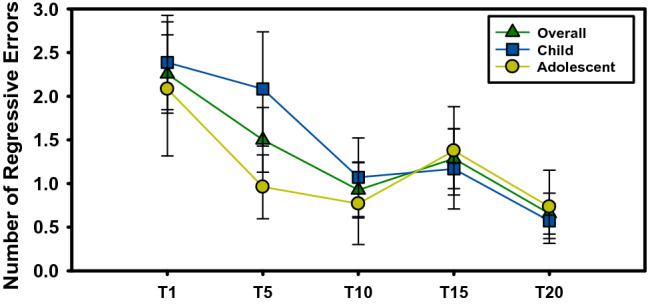
Number of regressive errors

For the overall sample (Table 1), there was a main effect of visit on trials to reach criterion [F(4,188) = 2.88, p = 0.02; Fig. 3; Table 2]. Planned post-hoc comparisons revealed non-significant practice effects from screen to baseline visits (p > 0.14), but significant reduction in trials to criterion from screen to treatment end (t = 2.44, p = 0.02), from screen to 10-week follow-up (t = 3.0, p = 0.003), from baseline (intervention start) to treatment end (t = 2.00, p = 0.04) as well as trending improvement from baseline to 10-week follow-up (t = 2.04, p = 0.06). Only the significant reduction in trials to criterion from screen to 10-week follow-up survived Bonferroni correction (p = 0.03). There was no main or interactive effects with Age Group (p’s > 0.42); however, as Fig. 3 demonstrates, there is clear marked difference in performance across visits for children versus adolescents. Children and adolescents demonstrated no differences in trials to criterion at screen (p = 0.82), but showed a trending difference at baseline [F(1, 177) = 3.61, p = 0.06]. With regard to errors, a main effect of Visit was found for regressive [F(4, 189) = 2.50, p = 0.04; Table 3] but not perseverative errors [F(4, 189) = 1.80, p = 0.39; Table 4]. A difference in performance in terms of regressive errors in children versus adolescents was observed across visits as seen for trials to criterion (Fig. 4).

**Table 1:** Demographic and clinical information of final sample

	ALL N = 29	CHILD (8–12 YR) N = 14	ADOLESCENT (13–18 YR) N = 15
Age	12.2 (2.8)	9.8 (1.6)	14.4 (1.5)***
Sex, %	26 (86)	12 (86)	13 (87)
FSIQ	97.4 (17.5) *69–130*	96.7 (18.7) *69–127*	98.1 (16.9) *71–130*
NVIQ	99.6 (15.9) *65–125*	95.2 (18.0) *65–125*	103.3 (13.5) *79–125*
VIQ	96.4 (18.5) *65–135*	97.5 (17.5) *77–123*	95.5 (20.0) *65–135*
ADOS SA CSS	8.2 (1.2) *6–10*	7.6 (1.0) *7–9*	8.5 (1.3) *6–10*
ADOS RRB CSS	4.9 (3.1) *0–9*	5.1 (3.8) *0–9*	4.8 (2.8) *0–7*
ADOS TOTAL CSS	7.6 (1.1) *6–10*	7.1 (0.9) *6–8*	7.8 (1.1) *6–10*
ABC-IRR	19.0 (9.0) *5–36*	21.1 (8.5) *9–36*	16.9 (9.4) *5–34*
ABC-HYPER	19.4 (11.9) *4–48*	21.8 (12.6) *7–48*	17.0 (11.1) *4–47*

Mean (standard deviation) and range in italics

*FSIQ* Full-scale IQ, *NVIQ* Non-verbal IQ, *VIQ* Verbal IQ, *ADOS* Autism Diagnostic Observation Scale, *SA* Social Affect, *CSS* Calibrated severity score, *RRB* Restricted, repetitive behavior, *ABC* Aberrant Behavior Checklist, *Irr* Irritability subscale, *Hyper* Hyperactivity subscale

***p > 0.001

**Table 2:** Trials to criterion across visits

	T1	T2	T3	T4	T5	p-value uncorrected*
Child	35.0 (13.2) *20–65*	35.8 (16.8) *20–100*	28.2 (10.3) *20–49*	29.2 (12.2) *19–63*	24.1 (4.3) *18–32*	T1 vs T20: p = 0.027 T5 vs T10: p = 0.098 T5 vs T20: p = 0.011

	N = 31	N = 24	N = 14	N = 12	N = 14	
Adolescent	34.8 (20.3) *20–100*	28.5 (2.1) *20–70*	26.2 (9.6) *20–54*	31.1 (15.1) *20–75*	27.1 (2.3) *20–50*	T1 vs T5: p = 0.101 T1 vs T10: p = 0.065 T1 vs T20: p = 0.027 T5 vs T10: p = 0.098 T5 vs T20: p = 0.011

	N = 24	N = 26	N = 13	N = 16	N = 15	
Overall	34.9 (9.6) *20–100*	32.0 (14.3) *20–100*	27.2 (13.7) *20–54*	30.3 (7.2) *20–75*	25.7 (7.2) *20–50*	T1 vs T10: p = 0.016 T1 vs T20: p = 0.003 T5 vs T20: p = 0.047
	N = 55	N = 50	N = 27	N = 28	N = 29	

Visits mean (SD) and range in italics

*Uncorrected p-values only provided for significant or trending post-hoc comparisons

**Table 3:** Regressive errors across visits

	T1	T2	T3	T4	T5	p-value uncorrected*
Child	2.4 (0.5) 0–11	2.1 (0.7) 0–12	1.1 (0.5) 0–5	1.2 (0.5) 0–5	0.6 (0.2) 0–2	T1 vs T20: p = 0.059 T5 vs T20: p = 0.071
Adolescent	2.1 (0.8) 0–13	1.0 (0.4) 0–8	0.8 (0.5) 0–6	1.4 (0.5) 0–6	0.7 (0.4) 0–5	T1 vs T20: p = 0.099
Overall	2.3 (0.4) 0–13	1.5 (0.4) 0–12	0.9 (0.3) 0 = 6	1.3 (0.3) 0–7	0.7 (0.2) 0–5	T1 vs T10: p = 0.024 T1 vs T15: p = 0.96 T1 vs T20: p = 0.006

*Uncorrected p-values only provided for significant or trending post-hoc comparisons

**Table 4:** Perseverative errors across visits

	T1	T2	T3	T4	T5	p-value uncorrected*
Child	1.3 (0.2) 0–4	1.6 (0.3) 0–6	1.4 (0.2) 1–4	1.0 (0.2) 0–2	1.3 (0.3) 0–4	n.s
Adolescent	1.5 (0.4) 0–7	1.5 (0.2) 0–6	1.2 (0.1) 1–2	1.2 (0.3) 0–5	1.9 (0.2) 1–4	n.s
Overall	1.4 (0.2) 0–7	1.6 (0.2) 0–6	1.3 (0.1) 1–4	(0.2) 0–5	1.6 (0.2) 0–4	n.s

*Uncorrected p-values only provided for significant or trending post-hoc comparisons

### Clinical Relationships

At screen, increased number of trials to criterion (r = − 0.51, p = 0.02) and number of regressive errors (r = − 0.52, = 0.01) was associated with lower IQ score. Screen and baseline parent-report clinical symptoms on the ABC did not relate to their corresponding visit performance-based variables. However, reduction in trials needed to reach criterion at week 10 follow-up relative to baseline was related to more severe ABC Irritability at baseline (r = − 0.40, p = 0.04).

## Discussion

We demonstrate the initial feasibility, reproducibility, and utility of a probabilistic reversal learning task as an outcome measure of cognitive flexibility following a within subjects trial of a group-based intervention in children and adolescents with ASD. Nearly all participants demonstrated successful completion of PRL across all study visits. We also observed participants needing fewer number of trials to reach PRL criterion and making fewer regressive errors following intervention compared to baseline, suggesting PRL is sensitive to detect treatment-related change in cognitive/behavioral flexibility. Together, we demonstrated feasibility, acceptability, and change sensitivity of PRL in individuals with ASD, implicating the need for future studies with larger samples to determine its utility as an outcome measure for interventions targeting behavioral inflexibility in this patient population.

With few interventions targeting cognitive flexibility in ASD and their limited efficacy based on available parent-report and performance-based outcome measures (de Vries et al., 2015; Fisher & Happé, 2005; Kenworthy et al., 2014; Yerys et al., 2019), the need for quantitative outcome measures of cognitive flexibility that are feasible to use and sensitive to change are critical for testing novel treatment strategies for this behavioral problem domain in ASD. Our pilot study demonstrates > 95% completion rate of PRL for youth with ASD ages 8–18 years. We did not show performance change between screen and baseline, even demonstrating good test/retest reliability (ICC > 0.6). This suggests that changes in behavioral performance observed following intervention were unlikely due to practice effects related to repeated testing and that scores were reproducible. In addition, we show youth with ASD needed fewer trials to reach criterion and improved ability to maintain new correct response once established (i.e., fewer regressive errors) immediately following intervention as well as at the 10-week follow-up.

Additionally, we observed inter-individual variability in amount of PRL performance change from baseline to post-intervention. This suggests PRL performance may not uniformly improve across participants, but rather, cognitive flexibility improved to varying degrees in the participants following *Regulating Together*. Indeed, we found that more severe irritability at baseline was associated with a greater reduction in regressive errors at follow-up. This suggests that participants with the most severe irritability coming into the study demonstrated the most improvement on the cognitive flexibility measure. It also is important to note that we only showed improvement on the number of regressive, as opposed to perseverative, errors. This suggests specificity of PRL as an outcome measure to identify specific aspects of cognitive flexibility improvement related to learning new behavioral choice preferences without returning to previous behavioral preferences, as opposed to being unable to shift response preferences at all, which has meaningful clinical implications.

Improved PRL performance was more marked in the Child age group (8–12 years) as compared to the Adolescent age group (13–18 years). These age-related effects on PRL outcome variables suggest children may be more likely to benefit in terms of cognitive flexibility from *Regulating Together*. This could be explained, in part, by the greater malleability of cognitive processes in children given their developmental maturation status. This finding should be considered in context of significant improvements in PRL performance also were found at the 10-week follow-up visit. This suggests performance is stable 10-weeks after the intervention concluded and the task is able to detect maintenance of therapeutic benefits to cognitive flexibility. Taken together, we not only demonstrate that PRL is sensitive to change in performance following intervention but also that improvements in cognitive flexibility are primarily present in children and maintained over time.

The current study has certain limitations. First, the current feasibility study occurred within the context of a pilot intervention study using a within-subjects design, limiting our sample size and ability to compare against an active control group. Though we did not observe practice effects from screen to baseline, it is possible change in performance is associated with long-term practice effects and not the intervention itself. Future studies using a randomized-control trial with a larger sample are needed to assess learning over multiple testing sessions, and to replicate behavioral performance findings. Second, the intervention trial excluded individuals with IQ < 60 and/or who did not have full sentence speech. Thus, feasibility findings for use of PRL as an outcome measure may be not be suitable for some very young individuals with emotional distress or for youth with ASD with co-occurring intellectual disability.

## Conclusion

Our preliminary findings from a within-subjects intervention trial of *Regulating Together* demonstrated the feasibility, reproducibility, and utility of a probabilistic reversal learning task as an outcome measure of cognitive flexibility youths with ASD. Nearly all participants (> 95%) successfully completed the task, implicating high feasibility and acceptability in a multi-visit trial. Furthermore, we found improvement in certain PRL variables from baseline, suggesting the measure may be sensitive to change in cognitive flexibility performance. Taken together, PRL should be considered a promising outcome measure of cognitive flexibility useful in intervention trials with youths with ASD.
